# A Paradoxical Evolutionary Mechanism in Stochastically Switching Environments

**DOI:** 10.1038/srep34889

**Published:** 2016-10-14

**Authors:** Kang Hao Cheong, Zong Xuan Tan, Neng-gang Xie, Michael C. Jones

**Affiliations:** 1 Engineering Cluster, Singapore Institute of Technology, 10 Dover Drive, Singapore 138683, Singapore; 2 Yale University, New Haven, CT 06520, United States; 3 Department of Mechanical Engineering, Anhui University of Technology, Anhui Ma’anshan, 243002, China; 4 Columbia, Missouri, United States

## Abstract

Organisms with environmental sensors that guide survival are considered more likely to be favored by natural selection if they possess more accurate sensors. In this paper, we develop a theoretical model which shows that under certain conditions of environmental stochasticity, selection actually favors sensors of lower accuracy. An analogy between this counter-intuitive phenomenon and the well-known Parrondo’s paradox is suggested.

All forms of cellular life depend on sensors to detect environmental cues that are then transduced to evoke an adaptive response, such as moving along a gradient of nutrients. Perfect sensors are difficult to evolve because the external environment always has some degree of unpredictability (extrinsic noise) and the molecular pathways of stimulus-response also have some degree of stochastic variability (intrinsic noise)^1^,^2^,^3^,^4^. The time delay between stimulus and response also means that accurate detection by a sensor may result in a non-optimal response if the environment changes during the delay period. As a result, organisms must cope with the uncertainty of the future with the best deterministic sensor they can evolve in combination with the best randomized guess that they can make. Such mixed deterministic-stochastic strategies are ubiquitous in nature^5^.

Many environmental variables fluctuate unpredictably, such as temperature, nutrient levels, and light intensity. In the presence of such fluctuations, selection may exploit the intrinsic randomness of biochemical reactions in order to switch phenotypes stochastically, ensuring that at least some individuals in a population make the right choice simply by chance^6^. By spreading the risk horizontally (spatially) across a population of individuals, the lineage may escape extinction. Stochastic phenotypic switching is an example of “evolutionary bet-hedging”^7^, also known as “adaptive coin-flipping”^8^ or “stochastic polyphenism”^9^. For instance, the “choice” by a small minority of pathogenic bacterial clones to become dormant in an actively growing and dividing population shields them from antibiotics^10^. Stochastic differences in seed germination times allow plants to survive unpredictable changes in rainfall or temperature^11^. Stochastic phenotypic decisions during insect^8^,^12^, amphibian^13^ and fish^14^ metamorphosis may allow survival in unpredictable wet/dry environments. At higher scales of complexity, the effect of stochastic phenotypic variance is important to realistic modeling of nonlinear interactions between evolving organisms and stochastic environments and how they influence ecosystem diversity, sustainability and evolution^10^,^15^,^16^,^17^,^18^.

To understand the evolution of stochastic phenotypic variance as a response to stochastic environments, much can be learned from bacteria. Individual clones may exhibit wildly unpredictable transitions between alternative states known as random phase variation (RPV). Random phase variation may be both temporal (across the life history of a single clone) and spatial (across a population of clones)^19^. Using a game-theoretic model of bacterial populations, Wolf *et al*. showed that under certain conditions, a mixed (stochastic/deterministic) strategy of bacterial RPV emerges as an evolutionarily stable strategy (ESS)^19^. Specifically, they found that if different environmental states select for different cell states, and if cells are unlikely to sense environmental transitions or are subject to long signal transduction delays relative to the time-scale of environmental change, then a time-varying environment can select for RPV. They also noted that the success of RPV can be understood as a variant of Parrondo’s paradox^20^, in which random alternations between losing strategies (in this case, cells that choose the wrong phase variation or sequence of variations) produce a winning strategy.

In this paper, we adapt the game theoretic population model used by Wolf *et. al*.^19^ in order to study the result of selection under environmental stochasticity. The main difference here is that, rather than focusing on RPV, we study organisms that make an idealized binary decision: they can migrate away from their present environment, or remain in place. We examine how this decision is affected by sensor accuracy and the rates of stochastic environmental switching. In addition to considering the non-absolute accuracy of the sensors, mutation between different levels of sensor accuracy is accounted for. Our model reveals a narrow “Goldilocks zone” in parameter space where Parrondo’s paradox also emerges. The relevance of this finding to higher-order biological phenomena is discussed.

## Population Model

To study the effects of stochastic environmental switching upon the growth and evolution of organisms, we use a discrete-time population model where each time unit is a single generation. During each generation, environmental conditions switch between a more favorable and less favorable state with a certain probability, and organisms can migrate in response to the environment that they detect themselves to be in. The different phases in a single generation are listed below in chronological order:

- **Migration** Organisms use their environmental sensors and identify the environment they are in. Sensors are correct with a certain probability. If an organism detects itself to be in an unfavorable environment based on the sensor output, then it migrates to the other environment. Otherwise, the organism stays put.
- **Switching** The two environments switch conditions at random with a certain probability. When a switch occurs, the environment which was initially unfavorable becomes favorable, and vice versa.
- **Growth** The organisms then stay in their current environment and undergo a cycle of births and deaths, including mutations between different levels of sensor accuracy. Note that even if an environmental switch occurred in the previous phase, organisms are not be able to migrate to another environment until the growth phase is complete. This reflects the asynchrony between environmental stimulus and organismal response that was noted in the introduction.

We now introduce the mathematical formulation of our model in greater detail.

### Environment

Environmental conditions affect the birth rate and death rate of organisms in that environment. As such, an environmental state *E*_*i*_, 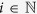 can be defined as function that maps environmental conditions like nutrient concentration and light intensity to a growth multiplier. That is, given an initial population size *x* and environmental conditions *ε*_*k*_, 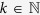, the population size *x*′ after a single generation is


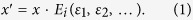


In our model, we consider only two environmental states *E*_1_ and *E*_2_. For simplicity of analysis, they are defined as the following constant multipliers:


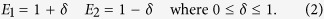


Here, *E*_1_ represents the favorable environment (a population in environment 1 will grow over time), *E*_2_ represents the unfavorable one (a population in environment 2 will shrink), and *δ* is a fixed constant that determines how favorable and unfavorable the environments are (respectively) compared to an environment where the growth rate is unity. To give an example, for a photosynthetic organism, *E*_1_ might correspond to a well-lit environment, while *E*_2_ might correspond to a dimly-lit environment. Switching between these environments could be due to stochastic variation in cloud cover or shade.

### Sensors

An organism relies upon environmental sensors to identify its current environmental conditions. In general, these sensors may be imperfect, and we model this by assigning a sensor accuracy level *s*_*j*_, 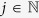, *j* ≤ *L*, to each organism, where *L* is the total number of sensor levels. With probability *s*_*j*_, the organism will correctly determine its environment in the beginning of the migration phase.

### Population

Since organisms in our population model can be in different environments and possess sensors with different accuracy levels, they can exist in a variety of states. We represent the sub-population in each state as *x*_*ij*_ ≥ 0, where *i* denotes the environment of the sub-population, and *j* denotes the *j*th sensor level. Since there are *L* sensor levels and 2 environments, the total population can be represented as a vector **X** of these sub-populations with length 2*L*:


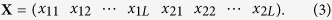


The total population size is thus given by the 1-norm of **X**:


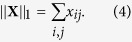


### Migration

During the migration phase, organisms migrate to a different environment if their sensors (which may be incorrect) detect that they are in the unfavorable environment (environment 2). Otherwise, they stay put. It follows that an organism in the sub-population *x*_1*j*_ will stay put with probability *s*_*j*_ (correct detection) and migrate to the sub-population *x*_2*j*_ with probability 1 − *s*_*j*_ (incorrect detection). On the other hand, an organism in the sub-population *x*_2*j*_ will migrate to the sub-population *x*_1*j*_ with probability *s*_*j*_ (correct detection) and stay put with probability 1 − *s*_*j*_ (incorrect detection). The expected post-migration sub-populations 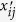 can then be computed in terms of the pre-migration sub-populations *x*_*ij*_:


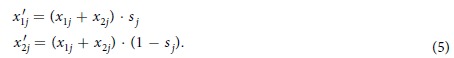


Using matrix notation, the process above can be expressed more concisely. We define the migration matrix **M** as a 2*L* × 2*L* matrix composed of four *L* × *L* diagonal matrices as follows:


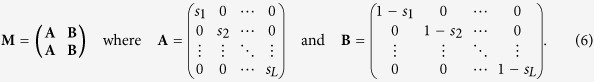


The *expected* value of the post-migration population vector is then given by


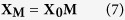


where **X**_**0**_ is the initial population vector.

In our model, it is assumed that the actual value of the population vector after migration is equal to the expected value, instead of drawing random values from a binomial distribution. This assumption is reasonable because the number of organisms is large enough that the standard deviation of the actual value from the expected value is very low (i.e. the law of large numbers applies).

### Switching

Every generation, the two environments switch conditions at random with a switching probability *p*. Switching can be represented by having all the organisms in environment 1 simultaneously migrate to environment 2, and vice versa. When switching occurs, the post-switching sub-populations 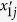 and 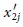 are given by


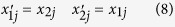


where *x*_1*j*_ and *x*_2*j*_ are the pre-switching sub-populations.

As with migration, the switching process can be concisely represented in matrix form. When the environment does not switch, the switching matrix is given by


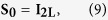


that is, it is just the 2*L* × 2*L* identity matrix. When the environment switches, the switching matrix is given by


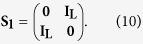


The population vectors after the switching phase are thus


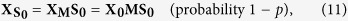



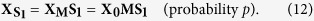


### Growth

In the growth phase, organisms undergo a cycle of births, deaths and mutations, resulting in an overall increase or decrease in population size. As explained earlier, the population of organisms in each environment grows at a rate dependent on the environmental conditions. We first consider the case with no mutation. The post-growth sub-population 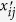 is given by


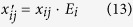


where *x*_*ij*_ is the pre-growth sub-population.

Mutation between different levels of sensor accuracy is also accounted for in order to study the effects of environmental stochasticity upon the evolution of environmental sensors. This is modelled by assigning a probability *m*_*ik*,*ij*_, *k* ≠ *j* to an organism in environment *i* and sensor state *k* birthing an organism in the same environment with sensor state *j*. Generalizing Equation 13 to take mutations into account gives


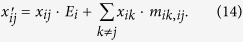



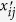
 here represents the *expected* value of the sub-population after growth and mutation. But as in the migration phase, we assume that the actual value is equal to the expected value given that we are simulating a large number of organisms.

As with the other phases, the growth phase can be expressed as a matrix **G**. For example, when we have *L* = 3 sensor levels, **G** is the following 6 × 6 matrix


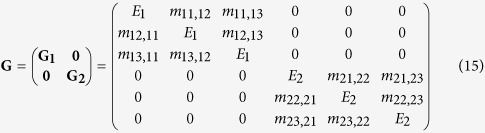


where **G**_**1**_ and **G**_**2**_ are the growth sub-matrices for environment 1 and 2 respectively. The population vectors post-growth are thus


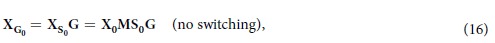



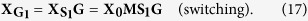


### Expected Population Vector

Note that the post-growth vectors are just the population vectors after one generation. Since there are two different possibilities (either the environment switches, or it does not), we can define a random variable 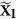 that represents the population vector after a single generation:


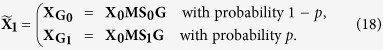


The expected population vector after a single generation is thus


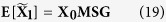


where **S** is the expected switching matrix


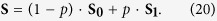


It can be shown that the expected population vector after *n* generations takes a similar form (see Proof A of the Supplementary Information):


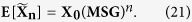


This allows us to easily analyse how the population vector changes on average over time.

### Mean Sensor Level

In addition to observing how the population vector changes over many generations, the evolution of the population can be studied by observing how the mean sensor level *μ* changes over time. *μ* can be defined as a function of the population vector **X**:


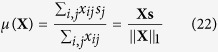


where **s** is the sensor level vector, defined as:


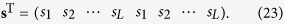


## Simulation Parameters

Simulations of our population model across a range of parameters revealed a paradoxical region of parameter space where accurate environmental sensors were unexpectedly selected against. For illustrative purposes, we provide a set of parameters where the paradoxical phenomenon was especially pronounced.

For environmental growth rates, the following values were used:


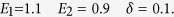


*L* = 3 imperfect sensor levels were used, with the values:


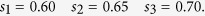


The population vector thus comprised 6 sub-populations. Each sub-population was assigned an initial value of 1000, giving the intitial population vector:


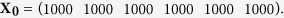


Two sets of simulations were performed to study the impact of mutation upon the evolution of the population. In the first set, all mutation rates were set to zero, thereby isolating all organisms with a particular sensor level *s*_*i*_ from other organisms with sensor levels *s*_*j*_, *j* ≠ *i*. In the second set, the following mutation rates were used:


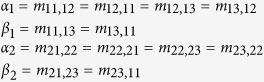



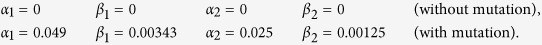


Here, *α*_*i*_ is the mutation rate in environment *i* between adjacent sensor levels (*s*_1_ and *s*_2_, or *s*_2_ and *s*_3_), while *β*_*i*_ is the mutation rate between distant sensor levels (*s*_1_ and *s*_3_) in the same environment. Note that *α*_1_ ≥ *α*_2_, and *β*_1_ ≥ *β*_2_, because mutation is more likely in environment 1 due to the higher birth rate.

Furthermore, *α*_*i*_ is greater than *β*_*i*_ because mutation between adjacent sensor levels is more likely to occur. For example, sensor accuracy might be a polygenic trait controlled by two gene loci. Each gene locus might have two alleles, one which results in a higher sensor accuracy, and the other which results in a lower accuracy. Mutation between distant sensor levels would only occur if both genes mutated simultaneously, making it less likely to occur than mutation between adjacent levels, which would only require a single gene to change.

Substituting the relevant variables into Equations 6, 9, 10 and 15 gives the migration, switching, and growth matrices:


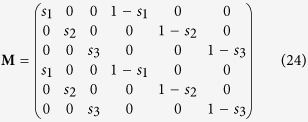



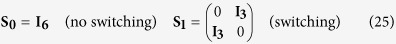



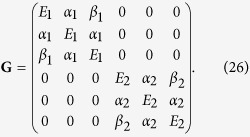


## Results

**Figure 1:**
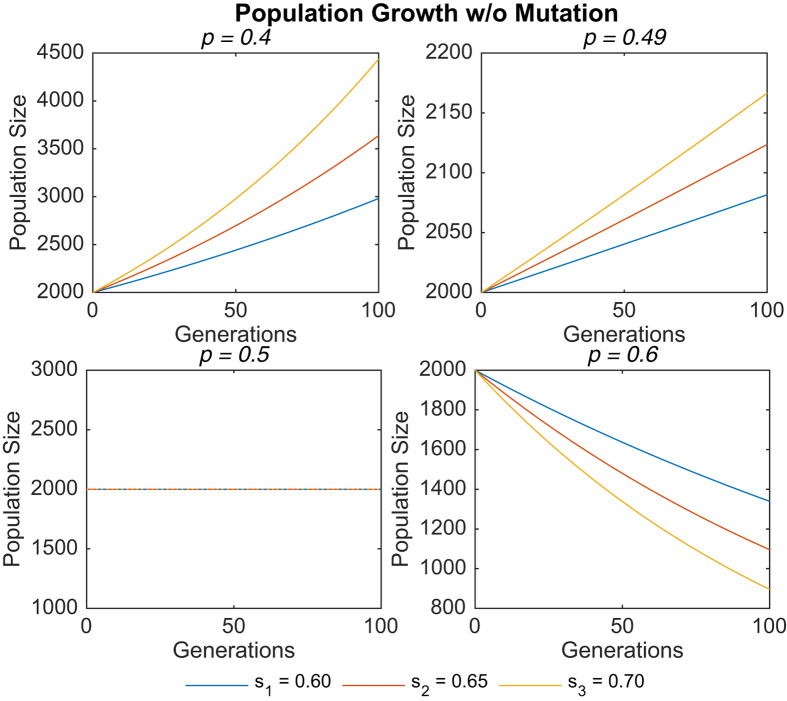
Population growth over time in the absence of mutation for different values of *p*. For *p* = 0.5, all three trend lines overlap.

**Figure 2:**
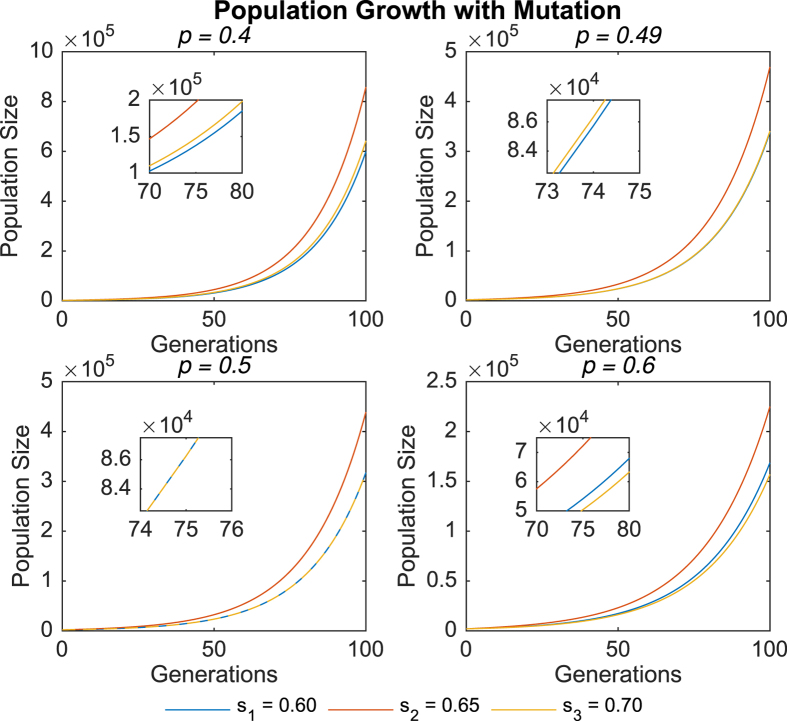
Population growth over time in the presence of mutation for different values of *p*. For *p* = 0.5, the trend lines for *s*_1_ and *s*_3_ overlap.

During the simulation, results were collected to study both population growth and the evolution of the mean sensor level *μ* over time. Simulation data were averaged over 10^6^ trials. Figures 1 and 2 show the population growth data for representative values of the switching probability *p*, in the absence and presence of mutation respectively.

Despite the large differences between the two sets of data, an important similarity should be noted: In both cases, when *p* < 0.5, the sub-population with sensor level *s*_3_ grew at a faster rate than the sub-population with sensor level *s*_1_. Conversely, when *p* > 0.5, the sub-population with sensor level *s*_3_ grew at a slower rate (or decreased at a faster rate) than the sub-population with sensor level *s*_1_. For clarity’s sake, only a few values of *p* are depicted, but this trend was seen to hold across all other switching probabilities simulated. Furthermore, the rates of population growth for *s*_1_ and *s*_3_ were equal when *p* = 0.5. A plausible interpretation of these results is that higher sensor levels are favored when *p* < 0.5, but disfavored when *p* > 0.5.

**Figure 3:**
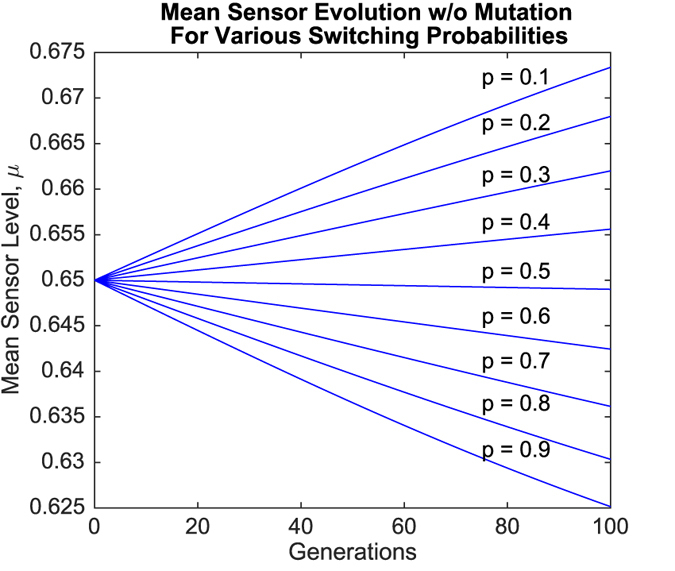
Evolution of mean sensor levels over time for different values of *p* in the absence of mutation.

**Figure 4:**
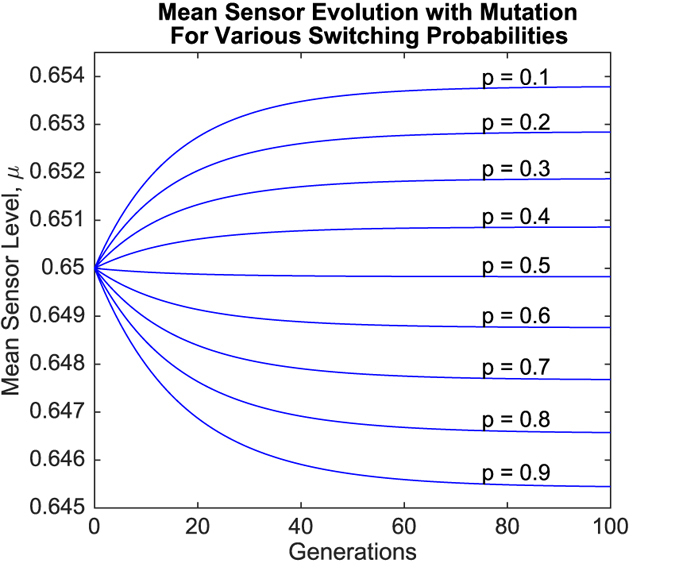
Evolution of mean sensor levels over time for different values of *p* in the presence of mutation.

Simulation results for the evolution of the mean sensor level *μ* are presented in Figs 3 and 4. Figure 3 depicts the results for various switching probabilities when mutation rates were zero, while Fig. 4 depicts the corresponding results when mutation is present.

Mutation exerted a stabilizing effect upon the evolution of *μ*, with convergence towards specific values of *μ* within the first 100 generations observed when mutation was present (refer to Fig. 4). Simulations also showed convergence of *μ* in the absence of mutation, but at much later times (no. of generations ≥ 400, not depicted) and only towards extremal values (*μ* approached either *s*_1_ = 0.60 or *s*_3_ = 0.70, or remained constant at *s*_2_ = 0.65).

**Figure 5:**
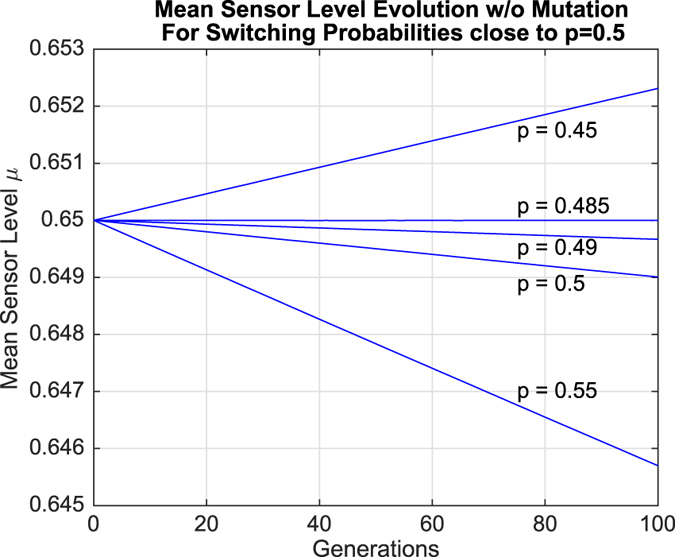
Evolution of mean sensor levels over time for values of *p* close to 0.5 in the absence of mutation.

**Figure 6:**
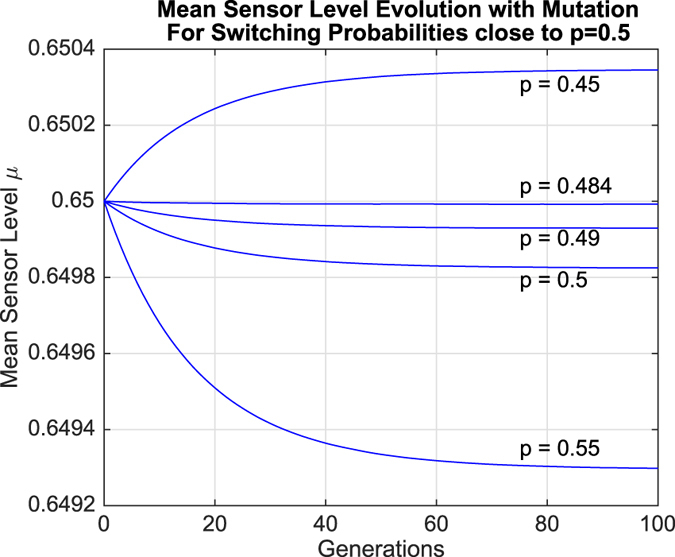
Evolution of mean sensor levels over time for values of *p* close to 0.5 in the presence of mutation.

Regardless of these effects, *μ* generally increased if *p* > 0.5 and decreased if *p* < 0.5. However, simulation results for values of *p* close to 0.5 revealed behavior that went against this general trend. These results are shown in Figs 5 and 6.

It can be observed in both sets of results that *μ* actually decreased when *p* = 0.5. In fact, there was a range of switching probabilities *p*_crit_ < *p* ≤ 0.5 within which *μ* decreased over time, where *p*_crit_ ≈ 0.485 when mutation was absent and *p*_crit_ ≈ 0.484 when mutation was present. In other words, higher sensor levels appear to be selected against within this region of parameter space. However, this contradicts the interpretation of the population growth data presented earlier, generating a paradox: mean sensor levels decrease in the region *p*_crit_ < *p* ≤ 0.5, even though organisms with sensor level *s*_3_ grow at a faster rate than organisms with sensor level *s*_1_. The following section explores this paradox in greater depth.

## Discussion

Table 1 summarizes the simulation results for both population growth (column 1) and the evolution of the mean sensor level *μ* (column 3). Additionally, based upon the observed results for population growth, the predicted trend for the evolution of *μ* is included in column 2. The paradoxical discrepancy between the predicted and observed trends for *μ* can be easily seen by comparing columns 2 and 3.

The results in column 1 are intuitively correct, given that the degree *δ* to which environment 1 is favorable to growth is equal to the degree to which environment 2 is unfavorable to growth. As a result, environmental conditions which are stable more than half the time (*p* < 0.5) favor the growth of organisms with better sensors because these organisms will on average migrate to the more favorable environment. Conversely, in conditions that are mostly unstable (*p* > 0.5), organisms with better sensors tend to migrate too aggressively for their own good, such that more often than not they end up in the unfavorable environment, causing their population to shrink faster.

Based upon the population growth data, a natural prediction to make would be that *μ* increases when *p* < 0.5, decreases when *p* > 0.5, and remains constant when *p* = 0.5. When *p* < 0.5, the organisms with sensor level *s*_3_ would grow to outnumber the organisms with sensor level *s*_1_, leading to the prediction that the mean sensor level increases. The opposite prediction would be made for *p* > 0.5, and when *p* = 0.5, the equal population sizes for organisms with sensor levels *s*_3_ and *s*_1_ should imply a constant level for *μ*. These predictions are listed in column 2.

However, the results in column 3 show otherwise. In particular, *μ* does not remain constant when *p* = 0.5, but at a specific critical probability *p*_crit_. The exact value of *p*_crit_ depends on the parameters (*p*_crit_ ≈ 0.485 without mutation and *p*_crit_ ≈ 0.484 based upon the data above), but it is clear from our simulations that *p*_crit_ < 0.5.

This gives rise to a paradoxical region *p*_crit_ < *p* < 0.5 within which *μ* decreases in spite of stable environmental conditions that favor sub-populations with better environmental sensors. To gain a better understanding of this counter-intuitive phenomenon, we first analytically derive the conditions for population growth in stochastically switching environments, showing that *p* = 0.5 is indeed the exact value below which organisms of sensor level *s*_3_ will grow more rapidly than those of sensor level *s*_1_. We then demonstrate that stronger growth in sub-populations with better sensors does not necessarily imply a corresponding increase in the mean sensor level, thereby resolving the paradox.

### Conditions for Population Growth

Consider a sub-population *y*_*j*_ = *x*_1*j*_ + *x*_2*j*_ of all the organisms with sensor level *s*_*j*_. The collection of these sub-populations across all *L* sensor levels can be written as a vector **Y**, given by


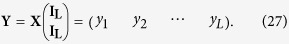


Using Equation 21, the expected **Y** vector after *n* generations is


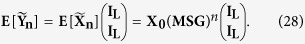


It can be shown that 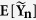 simplifies to


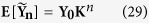


where 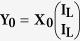 is the initial **Y** vector and **K** is the overall per-sensor-level growth matrix.

This gives a concise expression for the expected value of each *y*_*j*_ after *n* generations, through which the relative growth rate of each sensor state can be studied. When mutation is absent, the matrix **K** is particularly simple:


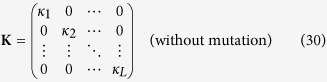


where the growth factor *κ*_*j*_ for sensor state *j* is given by


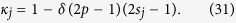


In other words, the expected value of *y*_*j*_ after *n* generations is just


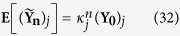


where (**Y**_**0**_)_*j*_ is the initial value of *y*_*j*_.

Clearly, this agrees with the simulation results. When both *s*_*j*_ > 0.5 and *p* > 0.5, *κ*_*j*_ < 1, resulting in a shrinking sub-population *y*_*j*_. On the other hand, when *s*_*j*_ > 0.5 but *p* < 0.5, *κ*_*j*_ > 1, resulting in the growth of *y*_*j*_. Furthermore, the larger the value of *s*_*j*_, the faster the rate of shrinking or growth.

When mutation is accounted for using the growth matrix in Equation 26, **K** takes on a more complicated form discussed in Proof B of the Supplementary Information. However, when *p* = 0.5, **K** simplifies to


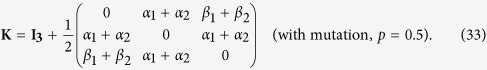


This gives a recursion relation for the expected values of *y*_1_ and *y*_3_ after *n* generations:


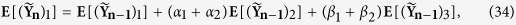



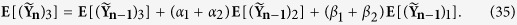


It follows that if initially *y*_1_ = *y*_3_, then both sub-populations will continue to grow at equal rates and maintain equal population sizes. This is precisely what was observed in the simulation results for *p* = 0.5. It can also be shown that *y*_1_ ≤ *y*_3_ if *p* < 0.5 and *y*_1_ ≥ *y*_3_ if *p* > 0.5, given these initial conditions. Proof C of the Supplementary Information contains a complete derivation of this result. The derivations of all the foregoing equations are also stated in the Supplementary Information for completeness. We conclude that regardless of mutation, *y*_3_ grows more quickly than *y*_1_ when *p* < 0.5, whereas *y*_1_ grows more quickly than *y*_3_ when *p* > 0.5.

### The Paradoxical Mechanism

As discussed earlier, even though *y*_3_ may grow more rapidly than *y*_1_ when *p* < 0.5, this does not simply imply that the mean sensor level *μ* will increase over time. As long as *p* > *p*_crit_, *μ* will decrease, even if *p* < 0.5. This paradoxical mechanism is best understood by analyzing how the population vector evolves over a single generation within the region *p*_crit_ < *p* < 0.5. For illustrative purposes, we study the case where mutation is present, and use *p* = 0.49. Suppose the initial population vector is the same as used in our simulation:


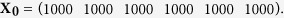


Initially, the mean sensor level of the population is


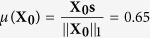


where the vector of sensor levels **s** is given by


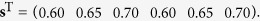


We can compute the post-growth population vectors (rounded to the nearest integer) using Equations 16 and 17:


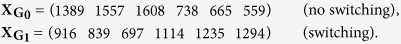


The expected population vector after one generation (rounded to the nearest integer) is thus


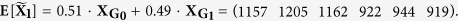


Computing the mean sensor level for 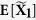 using Equation 22 (using the exact, un-rounded, values of the components) gives


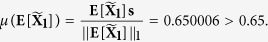


The sensor levels appear to *increase* from the initial value of 0.65. Since *p* < 0.5, this is in accordance with our earlier interpretation of the population growth data. However, it contradicts the simulated results for *p* = 0.49, which show that mean sensor levels should *decrease*.

This paradox can be resolved by understanding that the value we are concerned with is actually not 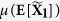, but rather 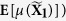. That is, instead of computing the mean sensor level of the expected population vector 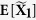, we should compute the expectation of the mean sensor level 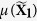 after a single generation. Since 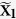 is a random variable, 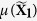 is also a random variable, given by


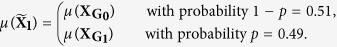


The expected value of 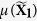 is then


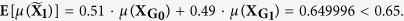


The mean sensor levels thus *decrease* after one generation, in accordance with the simulated results for *p* = 0.49. In general, it can be noted that for arbitrary *p* and *n*,


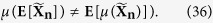


In other words, the mean sensor level of the expected population vector does not correspond to the expected sensor level. This is because computing the mean sensor level *μ* is a non-linear operation, and hence the order of operations matters. Note that despite the minuscule difference between the values of 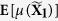 and of 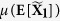 presented here, any such difference is still non-trivial, because it is all that is needed to explain the paradox.

It can be mathematically demonstrated that this phenomenon continues to occur at a higher number of generations, as well as for arbitrary *p* within the region of discrepancy. However, it is beyond the scope of this paper to derive a general trend as a function of all the parameters in our population model. This motivates future work.

### Stabilizing Effect of Mutation

One further observation that should be noted is that mutation results in stabilizing effect upon the evolution of the mean sensor level, *μ*. It can be seen in Fig. 4 that when mutation is present, *μ* ends up converging to a specific value between *s*_1_ = 0.60 and *s*_3_ = 0.70, with a different value of convergence for each switching probability *p*. However, when mutation rates are set to zero, *μ* does not converge within the first 100 generations, as can be seen in Fig. 3. Convergence still occurs, but much later in time (≥400 generations, not shown), and only to extremal values. In particular, when mutation is absent, *μ* converges to *s*_1_ = 0.60 when *p* > *p*_crit_, converges to *s*_3_ = 0.70 when *p* < *p*_crit_, and remains constant at *s*_2_ = 0.65 when *p* = *p*_crit_.

A natural way to understand this is that when mutation is absent, organisms with different sensor levels are effectively genetically isolated species. Setting mutation rates to zero thus models three different species of organisms in competition, with the result being the unbounded numerical dominance of one species over the others, and the convergence of *μ* towards an extreme value. However, when mutation is present at a certain level, the constant mutation between different sensor levels ensures continuous genetic diversity in the total population. Over time, this results in the emergence of a specific ratio between the different sub-populations as evolutionarily stable, and hence a constant value of *μ*.

### Analogy to Parrondo’s Paradox

Parrondo’s paradox^20^ is a phenomenon whereby two losing games, when played alternately in a random order, surprisingly end up winning. There have been many studies exploring the paradox^21^,^22^,^23^,^24^,^25^,^26^,^27^ and some involving population modeling and evolutionary biology^19^,^28^,^29^,^30^. Our results may possibly address the open problems involving Parrondo’s paradox in various evolutionary contexts^30^. For instance, in our model, the imperfect sensors are analogous to game B in the original paradox, and stochastic switching of the environment is analogous to the stochastic selection of the branches of game B. We have shown that under a favorable but narrow range of conditions of stochastic environmental switching, less accurate sensors are selected for, potentially demonstrating an evolutionarily stable strategy (ESS) that resists invasion by alternative strategies. That is, similar to Parrondo’s paradox, we expose a region of spontaneously emerging paradoxical behavior in which less accurate sensors are selected for over more accurate sensors, despite the more accurate sensors having a higher individual fitness.

Our simulated results predict that Parrondo’s paradox is likely to emerge only when the stochastic switching probability *p* is slightly less than 0.5. From Equation 31, we know that in this region, higher accuracy sensors have a higher individual fitness (higher growth factor), but only very slightly (difference on the order of 1 − 2*p*). We call this the “Goldilocks zone”. In this zone, stabilizing selection upon less accurate sensors results in their emergence as a potential ESS. Work in optimization theory has shown how multiple poor sensors can be combined to produce a more optimal, and hence more fit, sensor^31^, but in our case, the population actually evolves towards less absolute fitness when situated within the Goldilocks zone. In particular, the mean sensor level tends to decrease even though sensors with higher accuracy are still more fit than sensors with lower accuracy. The existence of a Goldilocks zone could thus jump start the evolution of poorer sensors in a population where better sensors are only very slightly favoured, which might potentially help explain how blindness emerged in organisms like eyeless cave fish and moles.

To explain the analogy in greater detail, we can use the characterization of Parrondo’s paradox as a convex linear combination of losing games. For the original paradox, it was shown that winning games could be constructed by picking certain linear combinations of losing games^27^. However, in our case, when *p* < 0.5, each sensor state *j* actually corresponds to a winning game (the growth factor *κ*_*j*_ is greater than one), and the mean sensor level *μ* corresponds to a convex linear combination of these games (since weighted means are convex linear combinations). What this study has shown is that within the Goldilocks zone, when different winning games (imperfect sensor states) play against each other, evolution drives the linear combination of these games in an unexpected direction: towards winning less, instead of winning more.

There is a possibility that any system situated within a Goldilocks zone could exhibit spontaneous, emergent behavior analogous to Parrondo’s paradox. Such behavior could then evolve into more sophisticated bet-hedging strategies involving stochastic phenotypic switching and polyphenic distributions, without requiring any systematic genetic differences between organisms. More specifically, once a ESS exhibiting Parrondo’s paradox is established, it may be subject to continuous directional selection that could drive it into more complex “losing game” architectures such as multicellularity and social systems of reproduction.

### Limitations

Our model limits the analysis to an idealized binary decision where an organism chooses between migrating or staying put. Future work will extend our model to the evolutionary trajectories of systems with more complex dimensionality, with potentially wide-ranging applications in evolutionary game theory and evolutionary bet-hedging.

## Conclusion

Our model has provided a surprising insight into the relationship between the accuracy of environmental sensors and evolutionary fitness. Even though organisms with more accurate sensors are intuitively expected to be more adaptive within stable environmental conditions, we find that mean sensor accuracy level of the entire population actually diminishes in quality when population size, mutation rates and the probability of stochastic environmental switching are in a narrow “Goldilocks zone” of parameter space.

This phenomenon is analogous, if not formally equivalent, to Parrondo’s paradox. Because this phenomenon emerges spontaneously within the “Goldilocks zone”, we hypothesize that it may potentially jump start the convergent evolution of poorer sensors across animal taxa, resulting in organisms like eyeless cave fish or mostly blind moles. It could also spark the evolution of altruistic systems of cooperative reproduction, since these altruistic strategies are “losing” strategies at the individual level. Exploring higher-dimensional extensions of our model to the evolution of cooperative systems is a goal of future work.

## Additional Information

**How to cite this article**: Cheong, K. H. *et al*. A Paradoxical Evolutionary Mechanism in Stochastically Switching Environments. *Sci. Rep*. **6**, 34889; doi: 10.1038/srep34889 (2016).

## Supplementary Material

Supplementary Information

## Figures and Tables

**Table 1:** Summary of observed and expected simulation results.

	Observed population growth	Predicted trend for *μ*	Observed trend for *μ*
*p* < 0.5	*s*_3_ growth rate > *s*_1_ growth rate	Increases with time	*p* < *p*_crit_: increases; *p* > *p*_crit_: decreases
*p* = 0.5	*s*_3_ growth rate = *s*_1_ growth rate	Stays constant	Decreases with time
*p* > 0.5	*s*_3_ growth rate < *s*_1_ growth rate	Decreases with time	Decreases with time

## References

[b1] Franciscis S., Caravagna G. & d’Onofrio A. Bounded Noises as a Natural Tool to Model Extrinsic Fluctuations in Biomolecular Networks. Natural Computing 13 **(3)**, 297–307 (2014).

[b2] Chalancon G. . Interplay Between Gene Expression Noise and Regulatory Network Architecture. Trends in Genetics 28 **(5)**, 221–232 (2012). 22365642 10.1016/j.tig.2012.01.006 PMC3340541

[b3] Caravagna G., Mauri G. & d’Onofrio A. The Interplay of Intrinsic and Extrinsic Bounded Noises in Biomolecular Networks. PLoS ONE 8 **(2)**, e51174 (2013). 23437034 10.1371/journal.pone.0051174 PMC3578938

[b4] Silva-Rocha R. & Lorenzo V. Noise and Robustness in Prokaryotic Regulatory Networks. Annual Review of Microbiology 64, 257–275 (2010). 10.1146/annurev.micro.091208.073229 20825349

[b5] Simons A. M. Fluctuating Natural Selection Accounts for the Evolution of Diversification Bet Hedging. Proceedings of the Royal Society of London B: Biological Sciences 276, 1987–1992 (2009). 10.1098/rspb.2008.1920 PMC2677257 19324774

[b6] Acar M., Mettetal J. & Oudenaarden A. Stochastic switching as a survival strategy in fluctuating environments. Nature Genetics 40, 471–475 (2008). 18362885 10.1038/ng.110

[b7] Seger J. & Brockmann J. What is Bet-hedging. Oxford Surveys in Evolutionary Biology 4, 182–211 (1987).

[b8] Cooper W. S. & Kaplan R. H. Adaptive “Coin-flipping”: A Decision-theoretic Examination of Natural Selection For Random Individual Variation. Journal of Theoretical Biology 94 **(1)**, 135–151 (1982). 7078205 10.1016/0022-5193(82)90336-8

[b9] Walker T. J. Stochastic Polyphenism: Coping with Uncertainty. The Florida Entomologist 69 **(1)**, 46–62 (1986).

[b10] Lohmar I. & Meerson B. Switching Between Phenotypes and Population Extinction. Physical Review E 84 **(5)**, 051901 (2011). 10.1103/PhysRevE.84.051901 22181438

[b11] Gremer J. R. & Venable D. L. Bet Hedging in Desert Winter Annual Plants: Optimal Germination Strategies in a Variable Environment. Ecology Letters 17 **(3)**, 380–387 (2014). 24393387 10.1111/ele.12241

[b12] Menu F. & Desouhant E. Bet-hedging for Variability in Life Cycle Duration: Bigger and Later-emerging Chestnut Weevils Have Increased Probability of A Prolonged Diapause. Oecologia (Berlin) 132 **(2)**, 167–174 (2002). 10.1007/s00442-002-0969-6 28547348

[b13] Kaplan R. H. & Cooper W. S. The Evolution of Developmental Plasticity in Reproductive Characteristics: An Application of the “Adaptive Coin-Flipping” Principle. The American Naturalist 123 **(3)**, 393–410 (1984).

[b14] Pinceel T. . Early and Late Developmental Arrest as Complementary Embryonic Bet-hedging Strategies in African Killifish. Biological Journal of the Linnean Society 114 **(4)**, 941–948 (2015).

[b15] Roques L. & Stoica R. Species persistence decreases with habitat fragmentation: an analysis in periodic stochastic environments. Journal of Mathematical Biology 55 **(2)**, 189–205 (2007). 17294236 10.1007/s00285-007-0076-8

[b16] Higgins K. Metapopulation Extinction Risk: Dispersal’s Duplicity. Theoretical Population Biology 76 **(2)**, 146–155 (2009). 19505487 10.1016/j.tpb.2009.05.006

[b17] Wennersten L. & Forsman A. Population-level Consequences of Polymorphism, Plasticity and Randomized Phenotype Switching: A Review of Predictions. Biological Reviews 87 **(3)**, 756–767 (2012). 22540928 10.1111/j.1469-185X.2012.00231.x

[b18] Gonzalez-Suarez M. & Revilla E. Variability in Life-history and Ecological Traits Is a Buffer Against Extinction in Mammals. Ecology Letters 16 **(2)**, 242–251 (2013). 23216830 10.1111/ele.12035

[b19] Wolf D., Vazirani V. & Arkin A. Diversity in times of adversity: probabilistic strategies in microbial survival games. Journal of Theoretical Biology 234, 227–253 (2005). 15757681 10.1016/j.jtbi.2004.11.020

[b20] Harmer G. & Abbott D. Losing strategies can win by Parrondo’s paradox. Nature(London) 402, 864 (1999).

[b21] Soo W. W. M. & Cheong K. H. Parrondo’s Paradox and Complementary Parrondo Processes. Physica A: Statistical Mechanics and its Applications 392 **(1)**, 17–26 (2013).

[b22] Soo W. W. M. & Cheong K. H. Occurrence of complementary processes in Parrondo’s paradox. Physica A: Statistical Mechanics and its Applications 412, 180–185 (2014).

[b23] Cheong K. H. & Soo W. W. M. Construction of novel stochastic matrices for analysis of Parrondo’s paradox. Physica A: Statistical Mechanics and its Applications 392 **(20)**, 4727–4738 (2013).

[b24] Ye Y., Wang L. & Xie N.-G. Parrondo’s games based on complex networks and the paradoxical effect. PLoS ONE 8, 1–11 (2013). 10.1371/journal.pone.0067924 PMC3699478 23844131

[b25] Abbott D. Asymmetry and disorder: A decade of Parrondo’s paradox. Fluctuations and Noise Letters 9 **(1)**, 129–156 (2010).

[b26] Flitney A. P. & Abbott D. Quantum models of Parrondo’s games. Physica A 324 **(1)**, 152–156 (2003).

[b27] Harmer G. P. & Abbott D. A review of Parrondo’s paradox. Fluctuation and Noise Letters 2 **(2)**, R71–R107 (2002).

[b28] Williams P. D. & Hastings A. Paradoxical Persistence Through Mixed-system Dynamics: Towards a Unified Perspective of Reversal Behaviours in Evolutionary Ecology. *Proceedings of the Royal Society of London B: Biological Sciences* **rspb20102074** (2011). 10.1098/rspb.2010.2074 PMC3061147 21270032

[b29] Reed F. A. Two-Locus Epistasis With Sexually Antagonistic Selection: A Genetic Parrondo’s Paradox. Genetics 176 **(3)**, 1923–1929 (2007). 17483431 10.1534/genetics.106.069997 PMC1931524

[b30] Harmer G. P., Abbott D., Taylor P. G. & Parrondo J. M. Brownian ratchets and Parrondo’s games. Chaos: An Interdisciplinary Journal of Nonlinear Science 11 **(3)**, 705–714 (2001). 10.1063/1.1395623 12779509

[b31] Challet D. & Johnson N. F. Optimal combinations of imperfect objects. Phys. Rev. Lett. 89, 028701 (2002). 12097021 10.1103/PhysRevLett.89.028701

