# Supplementary material for: A Paradoxical Evolutionary Mechanism in Stochastically Switching Environments
Source: Sci Rep. 2016 Oct 14;6:34889. doi: 10.1038/srep34889 (PMC5064378; doi:10.1038/srep34889)
Supplement: Supplementary Information [file srep34889-s1.pdf]

Supplementary Information:
A Paradoxical Evolutionary Mechanism in Stochastically Switching Environments

Kang Hao Cheong[1,*], Zong Xuan Tan[2], Neng-gang Xie[3], and Michael C. Jones[4]

[1]Engineering Cluster, Singapore Institute of Technology, 10 Dover Drive, Singapore 138683, Singapore
[2]Yale University, New Haven, CT 06520, United States
[3]Department of Mechanical Engineering, Anhui University of Technology, Anhui Ma'anshan 243002, China
[4]Columbia, Missouri, United States
[*]To whom correspondence should be addressed. E-mail: Kanghao.Cheong@SingaporeTech.edu.sg

The results in this paper are corroborated by both numerical simulations and analytical derivations. Detailed analytical derivations are given below for completeness.

Proof A

Let $\widetilde{\mathbf{X}}_\mathbf{n}$ be the random variable that represents the population vector after n generations. $\widetilde{\mathbf{X}}_\mathbf{n}$ can be expressed as

$$\widetilde{\mathbf{X}}_\mathbf{n} = \mathbf{X_0}\prod_{i=1}^{n}(\mathbf{M}\widetilde{\mathbf{S}}_i\mathbf{G}) \tag{S1}$$

where each $\widetilde{\mathbf{S}}_i$ is an independent random variable representing the switching matrix for generation i:

$$\widetilde{\mathbf{S}}_i = \begin{cases} \mathbf{S_0} & \text{with probability } 1-p\,, \\ \mathbf{S_1} & \text{with probability } p\,. \end{cases} \tag{S2}$$

We can express $\widetilde{\mathbf{X}}_\mathbf{n}$ recursively in terms of $\widetilde{\mathbf{X}}_\mathbf{n-1}$ as

$$\widetilde{\mathbf{X}}_\mathbf{n} = \widetilde{\mathbf{X}}_\mathbf{n-1}\mathbf{M}\widetilde{\mathbf{S}}_n\mathbf{G}\,. \tag{S3}$$

Since $\widetilde{\mathbf{X}}_\mathbf{n-1}$ and $\widetilde{\mathbf{S}}_n$ are independent random variables, it follows that:

$$\begin{aligned} \mathrm{E}[\widetilde{\mathbf{X}}_\mathbf{n}] &= \mathrm{E}[\widetilde{\mathbf{X}}_\mathbf{n-1}]\mathbf{M}\mathrm{E}[\widetilde{\mathbf{S}}_n]\mathbf{G} \\ &= \mathrm{E}[\widetilde{\mathbf{X}}_\mathbf{n-1}]\mathbf{MSG} \end{aligned}$$

where $\mathbf{S}$ is the expected switching matrix, as defined in Equation 20.

We proceed by induction on n and Equation 21 follows (restated here):

$$\mathrm{E}[\widetilde{\mathbf{X}}_\mathbf{n}] = \mathbf{X_0}(\mathbf{MSG})^n\,.$$

Proof B

For the following analysis, we define for convenience the overall growth matrix for a single generation, $\mathbf{T} = \mathbf{MSG}$, as well as the complementary probabilities $q = 1-p$ and $t_i = 1-s_i$.

$\mathbf{T}$ can be expressed in terms of $\mathbf{A}$ and $\mathbf{B}$ from Equation 6 and $\mathbf{G_1}$ and $\mathbf{G_2}$ from Equation 15:

$$\mathbf{T} = \begin{pmatrix} \mathbf{C} & \mathbf{D} \\ \mathbf{C} & \mathbf{D} \end{pmatrix} \quad \text{where} \quad \mathbf{C} = (q\mathbf{A}+p\mathbf{B})\mathbf{G_1} \quad \text{and} \quad \mathbf{D} = (p\mathbf{A}+q\mathbf{B})\mathbf{G_2}\,. \tag{S4}$$

Proof.

$$\begin{aligned} \mathbf{T} = \mathbf{MSG} &= \begin{pmatrix} \mathbf{A} & \mathbf{B} \\ \mathbf{A} & \mathbf{B} \end{pmatrix} \begin{pmatrix} q\mathbf{I_L} & p\mathbf{I_L} \\ p\mathbf{I_L} & q\mathbf{I_L} \end{pmatrix} \begin{pmatrix} \mathbf{G_1} & \mathbf{0} \\ \mathbf{0} & \mathbf{G_2} \end{pmatrix} \\ &= \begin{pmatrix} q\mathbf{A}+p\mathbf{B} & p\mathbf{A}+q\mathbf{B} \\ q\mathbf{A}+p\mathbf{B} & p\mathbf{A}+q\mathbf{B} \end{pmatrix} \begin{pmatrix} \mathbf{G_1} & \mathbf{0} \\ \mathbf{0} & \mathbf{G_2} \end{pmatrix} = \begin{pmatrix} \mathbf{C} & \mathbf{D} \\ \mathbf{C} & \mathbf{D} \end{pmatrix} \end{aligned}$$

□

From this, we can derive Equation 29, restated here:

$$\mathrm{E}[\widetilde{\mathbf{Y}}_{\mathbf{n}}] = \mathbf{Y_0}\mathbf{K}^n$$

where $\mathbf{K}$ can be expressed as

$$\mathbf{K} = \mathbf{C} + \mathbf{D}. \tag{S5}$$

Proof.

$$\begin{aligned}
\mathrm{E}[\widetilde{\mathbf{Y}}_{\mathbf{n}}] &= \mathbf{X_0}\mathbf{T}^n \begin{pmatrix} \mathbf{I_L} \\ \mathbf{I_L} \end{pmatrix} = \mathbf{X_0} \begin{pmatrix} \mathbf{C} & \mathbf{D} \\ \mathbf{C} & \mathbf{D} \end{pmatrix}^n \begin{pmatrix} \mathbf{I_L} \\ \mathbf{I_L} \end{pmatrix} \\
&= \mathbf{X_0} \begin{pmatrix} (\mathbf{C}+\mathbf{D})^n \\ (\mathbf{C}+\mathbf{D})^n \end{pmatrix} = \mathbf{X_0} \begin{pmatrix} \mathbf{I_L} \\ \mathbf{I_L} \end{pmatrix} (\mathbf{C}+\mathbf{D})^n = \mathbf{Y_0}\mathbf{K}^n
\end{aligned}$$

□

When mutation is absent, we have $\mathbf{G_1} = E_1\mathbf{I_L}$ and $\mathbf{G_2} = E_2\mathbf{I_L}$. Given this, we can derive Equations 30 and 31:

$$\mathbf{K} = \begin{pmatrix} \kappa_1 & 0 & \cdots & 0 \\ 0 & \kappa_2 & \cdots & 0 \\ \vdots & \vdots & \ddots & \vdots \\ 0 & 0 & \cdots & \kappa_L \end{pmatrix} \qquad \text{(without mutation)}$$

$$\kappa_j = 1 - \delta(2p-1)(2s_j-1)$$

Proof. Since $\mathbf{A}$, $\mathbf{B}$, $\mathbf{G_1}$ and $\mathbf{G_2}$ are all diagonal matrices when mutation is absent, so are $\mathbf{C}$, $\mathbf{D}$, and $\mathbf{K} = \mathbf{C}+\mathbf{D}$. Recall that the ith diagonal entries of $\mathbf{A}$ and $\mathbf{B}$ respectively are s_i and $t_i = 1 - s_i$. Computing the ith diagonal entry of $\mathbf{K}$, $\kappa_i = C_{ii} + D_{ii}$, we have

$$\begin{aligned}
\kappa_i &= (qs_i + pt_i)E_1 + (ps_i + qt_i)E_2 \\
&= (qs_i + pt_i)(1+\delta) + (ps_i + qt_i)(1-\delta) \\
&= qs_i + pt_i + ps_i + qt_i + \delta(qs_i + pt_i - ps_i - qt_i) \\
&= (q+p)(s_i+t_i) + \delta(q-p)(s_i-t_i) \\
&= 1 - \delta(2p-1)(2s_i-1)
\end{aligned}$$

. □

When mutation is present and the number of sensor levels $L = 3$, $\mathbf{K}$ can be expressed as

$$\mathbf{K} = \begin{pmatrix} \kappa_1 & \alpha_1^* & \beta_1^* \\ \alpha_2^* & \kappa_2 & \alpha_2^* \\ \beta_3^* & \alpha_3^* & \kappa_3 \end{pmatrix} \qquad \text{(with mutation)} \tag{S6}$$

where we define the overall growth and mutation rates

$$\kappa_i = 1 - \delta(2p-1)(2s_i-1) \tag{S7}$$

$$\alpha_i^* = \bar{\alpha} - \sigma_\alpha(2p-1)(2s_i-1) \tag{S8}$$

$$\beta_i^* = \bar{\beta} - \sigma_\beta(2p-1)(2s_i-1) \tag{S9}$$

and where α_i^* are β_i^* are defined using the averages and the standard deviations of the mutation rates

$$\bar{\alpha} = \frac{\alpha_1+\alpha_2}{2} \qquad \sigma_\alpha = \frac{\alpha_1-\alpha_2}{2}, \tag{S10}$$

$$\bar{\beta} = \frac{\beta_1+\beta_2}{2} \qquad \sigma_\beta = \frac{\beta_1-\beta_2}{2}. \tag{S11}$$

Proof. We define the matrices $\overline{\mathbf{G}}$ and $\mathbf{G}_\sigma$ as:

$$\overline{\mathbf{G}} = \frac{1}{2}(\mathbf{G_1} + \mathbf{G_2}) = \begin{pmatrix} 1 & \bar{\alpha} & \bar{\beta} \\ \bar{\alpha} & 1 & \bar{\alpha} \\ \bar{\beta} & \bar{\alpha} & 1 \end{pmatrix} \qquad \mathbf{G}_\sigma = \frac{1}{2}(\mathbf{G_1} - \mathbf{G_2}) = \begin{pmatrix} \delta & \sigma_\alpha & \sigma_\beta \\ \sigma_\alpha & \delta & \sigma_\alpha \\ \sigma_\beta & \sigma_\alpha & \delta \end{pmatrix}. \tag{S12}$$

We can thus write $\mathbf{G_1}$ as $\overline{\mathbf{G}} + \mathbf{G}_\sigma$ and $\mathbf{G_2}$ as $\overline{\mathbf{G}} - \mathbf{G}_\sigma$. It follows from $\mathbf{K} = \mathbf{C} + \mathbf{D}$ that

$$\begin{aligned}
\mathbf{K} &= (q\mathbf{A} + p\mathbf{B})(\overline{\mathbf{G}} + \mathbf{G}_\sigma) + (p\mathbf{A} + q\mathbf{B})(\overline{\mathbf{G}} - \mathbf{G}_\sigma) \\
&= (q\mathbf{A} + p\mathbf{B} + p\mathbf{A} + q\mathbf{B})\overline{\mathbf{G}} + (q\mathbf{A} + p\mathbf{B} - p\mathbf{A} - q\mathbf{B})\mathbf{G}_\sigma \\
&= \overline{\mathbf{G}} - (p - q)(\mathbf{A} - \mathbf{B})\mathbf{G}_\sigma \\
&= \begin{pmatrix} 1 & \bar{\alpha} & \bar{\beta} \\ \bar{\alpha} & 1 & \bar{\alpha} \\ \bar{\beta} & \bar{\alpha} & 1 \end{pmatrix} + (2p-1)\begin{pmatrix} 2s_1 - 1 & 0 & 0 \\ 0 & 2s_2 - 1 & 0 \\ 0 & 0 & 2s_3 - 1 \end{pmatrix}\begin{pmatrix} \delta & \sigma_\alpha & \sigma_\beta \\ \sigma_\alpha & \delta & \sigma_\alpha \\ \sigma_\beta & \sigma_\alpha & \delta \end{pmatrix} \\
&= \begin{pmatrix} \kappa_1 & \alpha_1^* & \beta_1^* \\ \alpha_2^* & \kappa_2 & \alpha_2^* \\ \beta_3^* & \alpha_3^* & \kappa_3 \end{pmatrix}.
\end{aligned}$$

□

Proof C

Substituting $p = 0.5$ into the general expression for $\mathbf{K}$ gives us Equation 33. As shown earlier, when $p = 0.5$, the sub-populations y_1 and y_3 grow at equal rates. We can now also see why y_1 grows more slowly than y_3 if $p < 0.5$ and why y_1 grows more quickly than y_3 if $p > 0.5$, even if $y_1 = y_3$ initially. Expanding the matrix notation in Equation 29 using $\mathbf{K}$ from Equation S6 gives us the following recurrence relations:

$$\mathrm{E}[(\widetilde{\mathbf{Y}}_\mathbf{n})_1] = \kappa_1 \mathrm{E}[(\widetilde{\mathbf{Y}}_{\mathbf{n-1}})_1] + \alpha_1^* \mathrm{E}[(\widetilde{\mathbf{Y}}_{\mathbf{n-1}})_2] + \beta_1^* \mathrm{E}[(\widetilde{\mathbf{Y}}_{\mathbf{n-1}})_3], \tag{S13}$$

$$\mathrm{E}[(\widetilde{\mathbf{Y}}_\mathbf{n})_3] = \kappa_1 \mathrm{E}[(\widetilde{\mathbf{Y}}_{\mathbf{n-1}})_3] + \alpha_1^* \mathrm{E}[(\widetilde{\mathbf{Y}}_{\mathbf{n-1}})_2] + \beta_1^* \mathrm{E}[(\widetilde{\mathbf{Y}}_{\mathbf{n-1}})_1]. \tag{S14}$$

From Equations S7 to S9, it is clear that when $p < 0.5$, κ_i, α_i^* and β_i^* all increase with increasing s_i, whereas when $p > 0.5$, κ_i, α_i^* and β_i^* decrease with increasing s_i. Since $s_3 > s_1$, it follows from the recurrence relations that even if y_3 and y_1 start off being equal, y_3 will be greater than y_1 in the next generation if $p < 0.5$, whereas the opposite will occur if $p > 0.5$.
